# Phytochemical Characterization of *Olea europea* Leaf Extracts and Assessment of Their Anti-Microbial and Anti-HSV-1 Activity

**DOI:** 10.3390/v13061085

**Published:** 2021-06-07

**Authors:** Ichrak Ben-Amor, Maria Musarra-Pizzo, Antonella Smeriglio, Manuela D’Arrigo, Rosamaria Pennisi, Hammadi Attia, Bochra Gargouri, Domenico Trombetta, Giuseppina Mandalari, Maria Teresa Sciortino

**Affiliations:** 1 Unit of Biotechnology and Pathologies, Higher Institute of Biotechnology of Sfax, University of Sfax, Sfax 3029, Tunisia; ichrak.benamor.etud@fss.usf.tn (I.B.-A.); hamadi.attia@gmail.com (H.A.); bochragargouri@yahoo.fr (B.G.); 2 Department of Chemical, Biological, Pharmaceutical and Environmental Sciences, University of Messina, Viale SS. Annunziata, 98168 Messina, Italy; mmusarrapizzo@unime.it (M.M.-P.); antonella.smeriglio@unime.it (A.S.); mdarrigo@unime.it (M.D.); rpennisi@unime.it (R.P.); domenico.trombetta@unime.it (D.T.); gmandalari@unime.it (G.M.)

**Keywords:** *Olea europea* L., LC-DAD-ESI-MS analysis, antimicrobial effect, antiviral activity, herpes simplex virus 1

## Abstract

Owing to the richness of bioactive compounds, *Olea europea* leaf extracts exhibit a range of health effects. The present research evaluated the antibacterial and antiviral effect of leaf extracts obtained from *Olea europea* L. var. *sativa* (OESA) and *Olea europea* var. *sylvestris* (OESY) from Tunisia. LC-DAD-ESI-MS analysis allowed the identification of different compounds that contributed to the observed biological properties. Both OESA and OESY were active against Gram-positive bacteria (MIC values between 7.81 and 15.61 μg/mL and between 15.61 and 31.25 μg/mL against *Staphylococcus aureus* ATCC 6538 for OESY and OESA, respectively). The antiviral activity against the herpes simplex type 1 (HSV-1) was assessed on Vero cells. The results of cell viability indicated that *Olea europea* leaf extracts were not toxic to cultured Vero cells. The half maximal cytotoxic concentration (CC_50_) values for OESA and OESY were 0.2 mg/mL and 0.82 mg/mL, respectively. Furthermore, both a plaque reduction assay and viral entry assay were used to demonstrate the antiviral activity. In conclusion, *Olea europea* leaf extracts demonstrated a bacteriostatic effect, as well as remarkable antiviral activity, which could provide an alternative treatment against resistant strains.

## 1. Introduction

Herbal medicine has been widely employed to treat or prevent diseases using plant extracts. It is part of alternative medicine, which is rich in therapeutic phytochemicals that may lead to the development of novel drugs [1,2]. Olive leaves are one of the most common, traditional herbal teas used amongst Mediterranean people to cure certain conditions [3]. For this reason, interest in the potential health benefits of olive leaves has increased amongst scientists across various fields. The olive tree (*Olea europaea* L.) is one of the most important fruit trees in Mediterranean countries, where they cover 8 million ha, accounting for almost 98% of the world crop [3]. In Tunisia, olive agriculture is one of the most important agricultural activities [4]. Antioxidant, hypoglycemic, antihypertensive, antimicrobial, and anti-atherosclerotic effects of olive leaves have been reported in various studies [5,6,7,8,9]. The olive leaves from *O. europaea* are rich in biophenols, such as oleuropein (Ole), verbascoside, ligstroside, tyrosol, and hydroxytyrosol [10]. These compounds have shown several biological activities, including antioxidant, antithrombotic, as well as skin photoprotective properties [10,11]. Furthermore, some of these compounds have demonstrated antimicrobial activity by inhibiting the growth of a wide variety of bacteria and fungi [12,13]. The antibacterial effect of olive leaves has been correlated with the presence of olive phenolic compounds, such as tyrosol (TyEDA) and hydroxytyrosol (HyEDA) [14]. *O. europaea* is known to contain a mixture of polyphenolic compounds, including oleuropein, oleuropein aglycone, elenolic acid, and hydroxytyrosol, which are readily absorbed and bioavailable. The biological activities of *O. europaea* are mainly derived from these compounds [15]. Fredrickson [16] demonstrated that *O. europea* has potent antiviral activities against the herpes virus, hepatitis virus, rotavirus, bovine rhinovirus, canine parvovirus, and feline leukemia virus. Indeed, the antiviral effect of olive extracts from Iran has been demonstrated against herpes simplex virus type 1 (HSV-1) in Vero cells [17]. Herpes simplex virus represents a persistent human pathogen that resides in infected hosts for their lifetime [18]. Indeed, following primary infection, the HSV-1 virus can undergo lytic infection in epithelial cells and latent infection in sensory neurons [18]. Since HSV infections are often subclinical, the infection is widely becoming one of the world’s most prevalent sexually transmitted infections (STIs) [19]. The infection can be serious in immuno-compromised hosts, and may involve the central nervous system, which, if left untreated, could be associated with 70% of mortalities [20]. Acyclovir is widely used for the treatment of primary and recurrent HSV-1 infections [21]. Based on this evidence, the present study aimed to prepare and compare two olive leaf extracts from *O. europea* var. *sativa* (OESA) and *O. europea* var. *sylvestris* (OESY) from Sfax (south-east of Tunisia) in order to characterize their chemical composition and investigate their antibacterial and antiviral properties. The antimicrobial potential of OESA and OESY was evaluated against a range of Gram-positive and Gram-negative bacteria and the yeast *Candida albicans*. The antiviral mechanism exerted by OESA and OESY extracts was assessed against HSV-1.

## 2. Materials and Methods

### 2.1. Chemicals

Formic acid and methanol were LC-MS grade and purchased from Merck (Darmstadt, Germany). Reference standards oleuropein, rutin, luteolin-7-*O*-glucoside, and apigenin-7-*O*-rutinoside were purchased from Extrasynthese (Genay, France), whereas luteolin-7-*O*-rutinoside was purchased from Merck (Darmstadt, Germany). Other chemicals were of analytic grade.

### 2.2. Sample Origin

The samples of the olive leaves used were collected during the month of October 2018 from Sfax, southeast of Tunisia. Our choice was directed towards the most important olive variety Chemlali, *Olea europea* L. var. *sativa* (OESA) and *Olea europea* var. *sylvestris* (OESY). 

### 2.3. Sample Preparation

OLSA and OLSY olive leaves were air-dried in the dark for three weeks, after which they were powdered by a mechanical grinder. The extraction was carried out using a mixture of water/ethanol (50:50, *v*/*v*) by simple maceration for 24 h with gentle stirring. Finally, the freeze-dried extracts were stored at 4 °C until further use.

### 2.4. Phytochemical Screening

#### 2.4.1. Total Phenols

The total phenol content was determined according to Smeriglio et al. [22]. Briefly, 50 μL of OESA, OESY (0.5–4.0 mg/mL), and gallic acid as the reference standard (75.0–600 μg/mL) were added to the Folin-Ciocalteu reagent (1:10 *v*/*v*) and brought to 1 mL with deionized water. After 3 min, 10% sodium carbonate (500 mL) was added, and the sample was left in the dark at room temperature (RT) for 1 h and mixed every 10 min. The absorbance was recorded at 785 nm by an UV–Vis spectrophotometer (Shimadzu UV-1601, Kyoto, Japan). The results were expressed as g of gallic acid equivalents (GAE)/100 g of dry extract (DE).

#### 2.4.2. Flavonoids

The flavonoid content was evaluated according to Smeriglio et al. [23]. Briefly, 200 μL of OESA, OESY (0.375–3.0 mg/mL), and rutin as the reference standard (0.125–1.0 mg/mL) were added to 2 mg/mL of AlCl_3_ (1:1, *v*/*v*) and brought to 1.6 mL with 50 mg/mL sodium acetate. After 2.5 h, the absorbance was recorded at 440 nm by an UV–Vis spectrophotometer (Shimadzu UV-1601, Kyoto, Japan). The results were expressed as g of quercetin equivalents (QE)/100 g DE.

### 2.5. LC-DAD-ESI-MS Analysis

The phytochemical analysis of OESA and OESY was carried out using an Agilent high-performance liquid chromatography system (HPLC 1100 series) equipped with an UV–Vis photodiode array (PDA-G1315) detector and an ion trap mass spectrometer detector (IT-6320). An electrospray ionization (ESI) source was used in full scan mode, monitoring the precursor ions between *m*/*z* 50 and *m*/*z* 1000 in negative polarity by using the following parameters: capillary voltage, 3.5 kV; drying gas temperature, 350 °C; nitrogen flow, 10 L/min; and nitrogen pressure, 50 psi. Data processing was carried out by Agilent 6300 Series Ion Trap LC/MS system software (version 6.2). The chromatographic separation was achieved by a Luna Omega PS C18 column (150 mm × 2.1 mm, 5 µm; Phenomenex, CA, USA) using solvent A (0.1% formic acid) and solvent B (methanol) as the mobile phase. The elution program was the following: 0–2 min, 5% B; 2–10 min, 25% B; 10–20 min, 40% B; 20–30 min, 50% B; 30–40 min, 100% B; 40–45 min, 5% B; and 45–60 min, 5% B. The flow rate was 0.3 mL/min, whereas the column temperature and the injection volume were 25 °C and 5 μL, respectively. UV–Vis spectra were recorded in the range of 190–700 nm, and chromatograms were acquired at 254, 280, 340, 370, and 520 nm. The acquisition wavelength chosen to show and compare the phytochemical profile of both extracts, at which all of the identified peaks were visible, was 254 nm. Peaks were identified by comparing the retention time, mass, and UV–Vis spectra with literature data and, when available, with reference standards (oleuropein, rutin, luteolin-7-*O*-glucoside, luteolin-7-*O*-rutinoside, apigenin-7-*O*-rutinoside).

### 2.6. Antimicrobial Assay

#### 2.6.1. Microbial Strains and Culture Conditions

A range of strains obtained from the University of Messina’s in-house culture collection (Messina, Italy) was used for the susceptibility studies: *Staphylococcus aureus* ATCC 6538, methicillin-resistant *S. aureus* ATCC 43300 (MRSA), 12 clinical isolates of *S. aureus* obtained from the pharynges (strains 26, 526, 531, 550, 808, 814), from duodenal ulcers (strains 8, 14), from hip prostheses (strains 3, 6, 32, 84), *Escherichia coli* ATCC 10536, *Pseudomonas aeruginosa* ATCC 9027, and *Candida albicans* ATCC 10231. All bacterial strains were grown in Mueller–Hinton Broth (MHB, Oxoid, CM0405) at 37 °C (18–20 h), whereas *C. albicans* was cultured in RPMI 1640 at 30 °C (24 h).

#### 2.6.2. Susceptibility Assays

The minimum inhibitory concentration (MIC), the minimum bactericidal concentration (MBC), and the minimum fungicidal concentration (MFC) of OESA and OESY were determined by the broth microdilution method, according to CLSI [24]. The tested concentrations ranged from 2000 to 1.9 μg/mL of either OESA or OESY dissolved in DMSO. The final concentration of DMSO did not exceed 1% in each sample. The MIC was defined as the lowest concentration that completely inhibited bacterial growth after 20 h. The MFC was defined as the lowest concentration that completely inhibited fungal growth after 48 h. The MBCs were determined by seeding 20 μL from clear MIC wells onto Mueller–Hinton agar (MHA, Oxoid) plates. The MBC was defined as the lowest extract concentration that killed 99.9% of the final inocula after 24 h incubation.

### 2.7. Antiviral Assay

#### 2.7.1. Cell Lines and Viruses

VERO cell lines (American Type Culture Collection) were propagated in minimal essential medium (EMEM) and supplemented with 6% fetal bovine serum (FBS) (Lonza, Belgium) at 37 °C under 5% CO_2_. The prototype HSV-1 (F) strain was kindly provided by Dr. Bernard Roizman (University of Chicago, Chicago, IL, USA). The HSV-1 viral stocks were obtained from cell-free supernatant of Vero cells infected with HSV-1. The recombinant virus HSV-1-VP26GFP expressing a GFP-tagged VP26 protein was propagated in Vero cells as previously described [25].

#### 2.7.2. Cell Proliferation Assay

The cell viability assay was performed as previously described [26]. Briefly, Vero cells were grown in 96-well plates and treated with different concentrations of OESA and OESY extracts (0.05 mg/mL, 0.1 mg/mL, 0.2 mg/mL, 0.4 mg/mL, 0.8 mg/mL, and 1 mg/mL) for 72 h. The cell viability was determined with a cytotoxicity bioassay kit (Lonza Group Ltd., Basel, Switzerland) according to the manufacturer’s instructions. The GloMax^®^ Multi Microplate Luminometer (Promega Corporation, 2800 Woods Hollow Road, Madison, WI, USA) in combination with the ViaLight™ plus cell proliferation and cytotoxicity bioassay kit was used to detect the emitted light intensity related to ATP degradation. The measured luminescence value was converted into the cell proliferation index (%) as previously reported [19].

#### 2.7.3. Plaque Reduction Assay

The antiviral activity was evaluated by plaque reduction assay. The Vero cells were seeded on 24-well plates and infected with the virus inoculum for 1 h at 37 °C with gentle shaking. The virus was diluted to yield 60 plaques/100 µL. A time-of-addition approach was used: (i) the virus inoculum was added on Vero cells and, after the incubation time, the monolayers were covered with a medium containing 0.8% methylcellulose in the presence of OESA and OESY extracts; and (ii) the virus inoculum was added on Vero cells pre-treated with OESA and OESY extracts and, after infection, the monolayers were covered with a medium containing 0.8% methylcellulose. The concentrations used for the antiviral assay were as follows: 0.1 mg/mL, 0.2 mg/mL, 0.4 mg/mL, 0.8 mg/mL, and 1 mg/mL. Acyclovir was included as the control at various concentrations (1, 10, and 20 µM). After three days, the cells were fixed, stained with crystal violet, and visualized with an inverted microscope (Leica DMIL, Nuloch, Germany) for plaque detection. After the incubation time, the inoculum was removed, and the monolayers were overlaid with Dulbecco’s Modified Eagle’s Medium containing 0.8% methylcellulose in the presence of the extracts. The plates were incubated at 37 °C with 5% CO_2_ for 72 h, and the plaques were visualized by staining the cells with crystal violet. 

#### 2.7.4. The Binding Assay

Vero cells (4 × 10^5^ cells/well) were cultured in 12-well plates and uninfected (mock) or infected with HSV-1 VP26GFP virus at a multiplicity of infection (MOI) of 0.5 PFU/cell. The infection was carried out by pre-treating the cells and the viral suspension with 0.1 mg/mL of OESA and 0.2 mg/mL of OESY, respectively, for 1 h. After the incubation time, the virus was adsorbed on pre-treated cells for 1 h at 4 °C. The infection was carried out at 4 °C to allow only the binding of the virus, but not the entry into the cells during the infection step. The virus inoculum was then removed, and the monolayers were incubated with a medium containing the extracts. Medium alone or containing DMSO were used as the controls.

### 2.8. Western Blot Analysis and Antibodies

Cellular proteins were extracted from Vero cells using SDS sample buffer 1× (62.5 mM of Tris-HCl, pH = 6.8; Dithiothreitol (DTT) 1 M; 10% glycerol; 2% SDS; 0.01% Bromophenol Blue), and immunoblot analysis was performed using an equal quantity of proteins. The proteins were revolved on SDS 10% polyacrylamide gel electrophoresis (PAGE) and transferred to nitrocellulose membranes (BioRad Life Science Research, Hercules, CA, USA). The membranes were probed overnight at 4 °C with specific antibodies to detect GFP-VP26 protein. Specific proteins were detected with a secondary anti-mouse antibody linked to horseradish peroxidase (HRP). GAPDH was used as a loading control. The chemiluminescence was detected by using Western HRP substrate (Merk, Millipore, Burlington, MA, USA). Immunoblot band intensity was quantified by densitometry analysis using the TINA software (version 2.10, Raytest, Straubenhardt, Germany). Anti-GFP (sc-9996) and Anti-GAPDH (sc-32233) antibodies were purchased from Santa Cruz Biotechnology (Santa Cruz, CA, United States). Goat anti-mouse immunoglobulin G (IgG) antibody-HRP conjugate was purchased from Merk, Millipore.

### 2.9. Viral DNA Extraction and Real-Time PCR Analysis

Cellular DNA was extracted from TRIzol-lysed cells according to the manufacturer’s instructions (Life Technologies, Carlsbad, CA, USA). Briefly, the DNA solution was precipitated from the interphase and organic phase with 100% ethanol. The DNA pellet was washed twice with 0.1 M of sodium citrate in 10% ethanol and dissolved in 8 mM of NaOH. Real-time PCR analysis was performed by using a specific TaqMan HSV-1 probe in a Cepheid SmartCycler II System (Cepheid Europe, Maurens-Scopont, France); 1 µg of DNA template was mixed with 1 µM of deoxyribonucleotide triphosphate (dNTP) mix, 0.5 μM of forward and reverse primers, 1 µM of TaqMan probe, 1× NH_4_ reaction buffer, 2 mM of MgCl_2_, and 5 U/µL of DNA polymerase BIOTAQ (BIO-21040 Bioline) in a total volume of 25 µL. The oligonucleotide primers used were: HSV-1 Fw 5′-catcaccgacccggagagggac; HSV-1 Rev 5′gggccaggcgcttgttggtgta, HSV-1 TaqMan probe 5′-6FAM-ccgccgaactgagcagacacccgcgc-TAMRA, (6FAM is 6-carboxyfluorescein and TAMRA is 6-carboxytetramethylrhodamine). The amplification was performed as follows: (i) 10 min at 95 °C, (ii) 30 s at 95 °C for 40 cycles, (iii) 30 s at 55 °C, (iv) 30 s at 72 °C, and (v) 5 min at 72 °C. A negative sample was used as the amplification control for each run. The quantitation of HSV-1 DNA was generated using GAPDH as a housekeeping gene with the comparative Ct method. DNA extracted from Vero cells infected and treated with Acyclovir (20 µM) was used as a positive control. 

### 2.10. Statistical Analysis

The statistical analysis was performed with GraphPad Prism 8 software (GraphPad Software, San Diego, CA, USA) by using one-way analysis of variance (ANOVA). The significance of the *p*-value was indicated with asterisks (*, **, ***, ****), which indicate the significance of the *p*-value less than 0.05, 0.01, 0.001, 0.0001 respectively. The half maximal cytotoxic concentration (CC_50_) and the half maximal effective concentration (EC_50_) values were calculated by using non-linear regression analysis.

## 3. Results

### 3.1. Phytochemical Analyses

**Figure 1:**
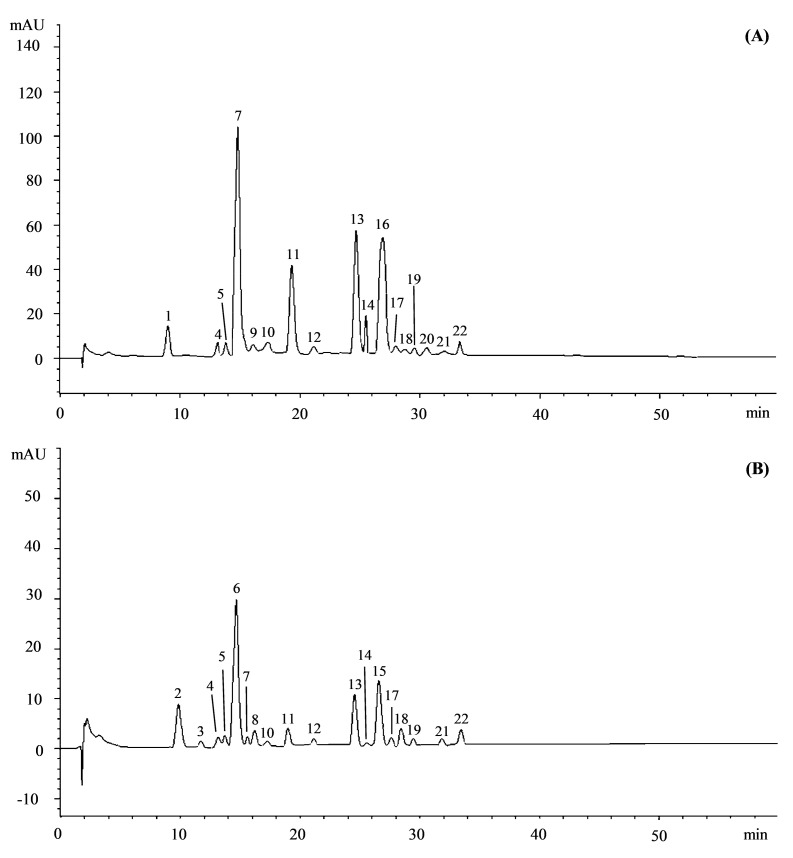
Representative LC-DAD chromatograms of *O. europea* L. var. *sativa* (OESA, panel **A**) and *O. europea* var. *sylvestris* (OESY, panel **B**) leaf hydroalcoholic extracts acquired at 254 nm. Peak numbers refer to the compounds listed in Table 1.

Preliminary phytochemical screening showed that the leaf hydroalcoholic extract of *O. europea* var. *sativa* (OESA) showed a higher content of total phenols (5.04 ± 0.29 g GAE/100 g DE vs. 4.40 ± 0.38 g GAE/100 g DE) and flavonoids (35.25 ± 3.19 g QE/100 g DE vs. 31.10 ± 1.81 g QE/100 g DE) with respect to the leaf hydroalcoholic extract of *O. europea* var. *sylvestris* (OESY). Comparative RP-LC-DAD-ESI-MS analysis of OESA and OESY extracts (Figure 1) revealed the presence of many polyphenols, characteristic of olive leaves. 

Seventeen and eighteen compounds were identified in the OESA and OESY leaf extracts, respectively. Table 1 shows the phytochemical profile of both extracts by listing the compounds according to their elution order.

**Table 1:** Phytochemical profile of the leaf hydroalcoholic extract of *O. europea* L. var. *sativa* (OESA) and *O. europea* var. *sylvestris* (OESY) by LC-DAD-ESI-MS analysis.

Peak n.	Compound	RT ^1^	λ_max_	[M-H]^−^	MW ^2^	Area % ^3^	
						OESA	OESY
1	Dihydroxybenzoic acid hexoside pentoside	9.083	296	447	448	4.95	-
2	Valoneic acid dilactone	9.9	256;365	469	470	-	8.69
3	Dicaffeoylquinic acid	11.7	218;327	515	516	-	5.87
4	Gallagic acid	13.309	234;296	603	604	0.97	10.38
5	Diellagilactone	13.831	252;377	601	602	1.00	0.89
6	Decaffeoylverbascoside	14.886	236;280	461	462	-	23.07
7	Oleoside/Secologanoside	14.955	244	389	390	32.68	0.94
8	Epicatechin 3-p-hydroxybenzoate	16.199	282;318	409	410	-	2.55
9	Elenoic acid hexoside	16.230	238	403	404	0.73	-
10	Hydroxyoleuropein isomer I	17.431	232;282	555	556	1.06	2.94
11	Oleanolic acid	19.461	232	454	455	11.48	1.91
12	Hydroxyoleuropein isomer II	21.390	232;282	555	556	0.58	3.75
13	Luteolin-7-*O*-rutinoside	24.821	258;344	593	594	13.86	9.84
14	Rutin	25.560	256;358	609	610	1.99	0.80
15	Oleuropein hexoside	26.855	232;282	701	702	-	11.07
16	Oleuropein	26.950	232;286	539	540	18.03	-
17	Apigenin-7-*O*-rutinoside	27.421	252;336	577	578	1.90	2.26
18	Luteolin-7-*O*-glucoside	28.326	268;342	447	448	6.14	6.92
19	Hydroxyphloretin 2′-*O*-xylosylglucoside	29.455	250;340	583	584	0.74	1.29
20	Vitisin A	30.440	388;510	560	561	0.76	-
21	β-Sitosteryl ferulate	32.140	240;294;318	590	591	0.84	0.56
22	Lucidumoside C	33.500	240;284	583	584	2.30	6.27

^1^ RT, Retention time; ^2^ MW, Molecular weight; ^3^ Results were expressed as mean area percentage of three independent experiments (*n* = 3) and is calculated with respect to the total peaks found.

The numbers refer to the peaks shown in Figure 1. Secoiridoids represented the most abundant class of compounds identified in OESA (55.38%), followed by flavones (21.90%), terpenoids (11.48%), and phenolic acids (4.95%). Minor compounds identified were other flavonoids (2.73%), ellagitannins (1.97%), phytosterols (0.84%), and stilbenoids (0.76%). On the contrary, in OESY, the content of secoiridoids (24.97%) was almost comparable to the content of phenylethanoids (23.07%), followed by flavones (19.02%), ellagitannins (11.27%), hydrolysable tannin (8.69%), and phenolic acids (5.87%). Minor compounds identified were other flavonoids (4.64%), terpenoids (1.91%), and phytosterols (0.56%). Bold numbers in Table 1 refer to the most abundant compounds identified in both leaf hydroalcoholic extracts. Oleoside/secologanoside (32.68%) was the most abundant compound identified in the OESA, followed by oleuropein (18.03%), luteolin-7-*O*-rutinoside (13.86%), oleanolic acid (11.48%), and luteolin-7-*O*-glucoside (6.14%). On the contrary, decaffeoylverbascoside (23.07%) was the most abundant compound identified in OESY, followed by oleuropein hexoside (11.07%), gallagic acid (10.38%), luteolin-7-*O*-rutinoside (9.84%), and valoneic acid dilactone (8.69%).

### 3.2. Antimicrobial Potential

The MIC values of OESA and OESY against the tested strains are shown in Table 2 and Table 3. Both extracts were active against the Gram-positive ATCC strains included in the study (MIC values between 7.81 and 15.62 μg/mL and between 15.62 and 31.25 μg/mL for OESY and OESA, respectively), whereas no activity was found against the Gram-negative bacteria or the yeast. As expected, the MIC values were lower for *S. aureus* ATCC 6538, demonstrating the higher sensitivity of this strain compared to the MRSA strain. The activity was always bacteriostatic rather than bactericidal. Amongst the clinical isolates, two strains were sensitive to both extracts, OESY being more active than OESA. The clinical isolates used in this study were previously characterized in relation to their antibiotic resistance [27]. Interestingly, strain 808 was sensitive to all antibiotics tested, with the exception of benzyl penicillin, whereas strain 6 was resistant to oxacillin, but sensitive to cefoxitin. As expected, the bacteriostatic inhibition of the clinical isolates was achieved at a higher concentration of both extracts compared with the standard strains.

The comparison between the two extracts showed a greater antimicrobial potential exerted by OESY rather than OESA, reflecting the different phytochemical composition of the two extracts (Table 1). This trend did not seem to relate to the total phenol content, which was higher in OESA. However, the phytochemical profile of the two extracts may be responsible for the different antimicrobial effect: secoiridoids were the most abundant class of compounds in OESA (55.38%), followed by flavones (21.90%) and terpenoids (11.48%), whereas the content of secoiridoids (24.97%) was almost comparable to phenylethanoids (23.07%) in OESY, followed by flavones (19.02%) and ellagitannins (11.27%).

### 3.3. Cytotoxicity of Olive Leaf Extracts on Cell Cultures 

**Figure 2:**
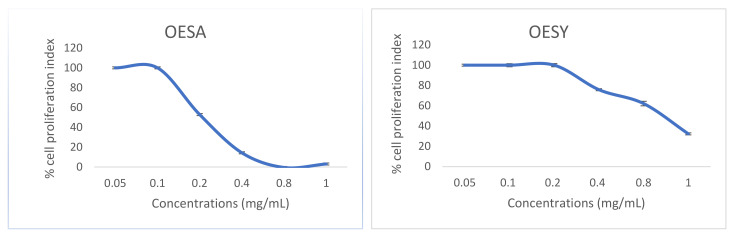
Viability assay in Vero cells treated with olive leaf extracts OESA and OESY. Vero cells were treated with different concentrations of olive leaf extracts (0.05 mg/mL, 0.1 mg/mL, 0.2 mg/mL, 0.4 mg/mL, 0.8 mg/mL, 1 mg/mL). The cells were collected 72 h post-treatment, and the luminescence value was converted into the cell proliferation index (%) as described in the Materials and Methods section. The assay was performed as the means of triplicates ± SD. OESY, extract of *O. europea* var. sylvestris; OESA, extract of *O. europea* var. sativa.

In order to examine the cytotoxicity effect of olive leaf extracts on Vero cells, we incubated the cells in the presence of different concentrations of OESA and OESY extracts for 72 h. Samples were then collected, and the quantification of the emitted light intensity related to ATP degradation was measured. Based on these results, the CC_50_ values were 0.2 mg/mL and 0.82 mg/mL for OESA and OESY, respectively (Figure 2).

### 3.4. Antiviral Activity of OESA and OESY

**Figure 3:**
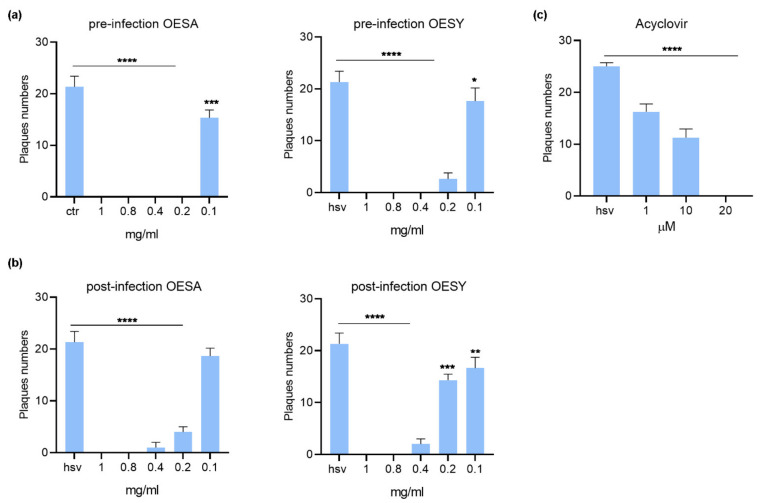
Plaque reduction assay from time-of-addition assay: (**a**) pre-infection condition, (**b**) post-infection condition treatments, and (**c**) Acyclovir control. Results are the mean ± SD of triplicate experiments, and asterisks indicate a significant *p*-value (* *p* < 0.05, ** *p* < 0.01, *** *p* < 0.001 and **** *p* < 0.0001). OESY, extract of *O. europea* var. sylvestris; OESA, extract of *O. europea* var. sativa.

The antiviral effect of OESA and OESY was determined against herpes simplex virus type 1 (HSV-1) using the plaque reduction assay. A time-of-addition assay was used. For the pre-infection treatment assay, Vero cells were treated with the extracts for 1 h and then infected with HSV-1 and incubated for 1 h at 37 °C. After the incubation time, the inoculum was removed, and the monolayers were overlaid with a medium containing 0.8% methylcellulose. For the post-infection treatment assay, Vero cells were infected with HSV-1 and incubated for 1 h at 37 °C. After the incubation time, the inoculum was removed and the monolayers were overlaid with a medium containing 0.8% methylcellulose in the presence of ethanolic extract of the leaf of OESA and OESY. The plates were incubated at 37 °C and 5% CO_2_ for 72 h, and the plaques were visualized by staining cells with crystal violet. Acyclovir was used as a control. The results showed a dose-dependent antiviral activity for both OESA and OESY (Figure 3). However, as indicated in Table 3, the selectivity index (SI) was higher for OESY (SI between 4.1 and 7.4) compared to OESA (SI between 1.3 and 1.6), and the pre-infection treatment condition was found to be more effective compared to the post-infection treatment for both types of extracts (Table 4).

### 3.5. OESA and OESY Prevent the Binding of HSV-1 on Vero Cells

**Figure 4:**
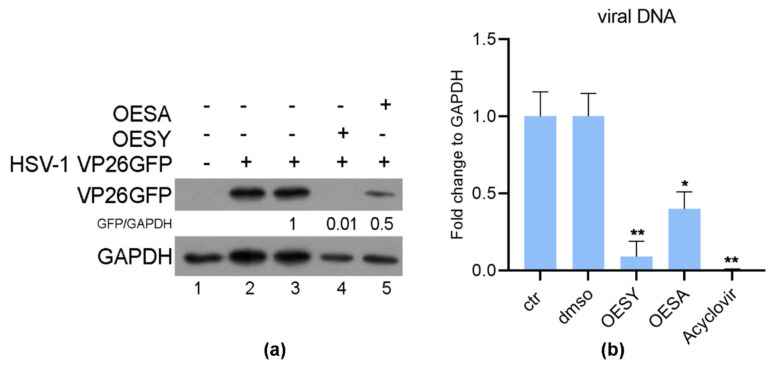
OESA and OESY prevent the binding of HSV-1. Vero cells infected or uninfected with HSV-1 VP26GFP, as described in the Materials and Methods section. Samples were processed 24 h p.i. for western blot analysis (**a**) and real-time PCR (**b**). DNA extracted from Vero cells infected and treated with Acyclovir (20 µM) was used as a positive control for the quantization of viral DNA. Data are expressed as the mean (± SD) of at least three experiments, and asterisks (* and **) indicate the significance of *p*-values less than 0.05 and 0.01, respectively. OESY, extract of *O. europea* var. *sylvestris*; OESA, extract of *O. europea* var. *sativa*.

The results obtained from the plaque reduction assay indicated that the SI was higher when the cells were pre-treated with the extracts before infection. To confirm the hypothesis that the binding of the virus could be prevented by both extracts, a binding inhibition assay was performed as indicated in the Materials and Methods section. The binding inhibition assay was carried out by using a recombinant HSV-1-VP26GFP virus. The infection was carried out at 4 °C to allow the binding of the virus to the cell receptors, but not the entry. Twenty-four hours post-infection, the expression of the VP26GFP protein was detected by western blot analysis, and quantitative real-time PCR was carried out to quantify the viral DNA. DNA extracted from Vero cells infected and treated with Acyclovir (20 µM) was used as a positive control for the quantization of viral DNA. As shown in Figure 4, pretreatment of cells and the virus with either OESA (0.1 mg/mL) or OESY (0.2 mg/mL) was able to reduce the accumulation of VP26 viral protein, as well as the accumulation of viral DNA, compared to the DMSO control. Moreover, OESY showed a greater antiviral activity compared to OESA, confirming the previous results obtained in Figure 3.

## 4. Discussion

Plant extracts and pure compounds have been widely tested for their antioxidant and antimicrobial activity. Olive leaves have very good biological activities, such as antihypertensive, anti-atherogenic, anti-inflammatory, hypoglycemic, and hypocholesterolemic effects [28,29]. In the present study, the RP-LC-DAD-ESI-MS analysis revealed the presence of many polyphenols in OESA and OESY extracts. The obtained results indicated luteolin- and apigenin-7-*O*-glycosides to be the predominant flavonoids in olive leaves, followed by rutin. These data are in accordance with previous observations carried out in olive leaf extracts obtained from five cultivars grown in southern Spain [10], as well as Portuguese olive cultivars [30]. Moreover, Pereira and coworkers [3] identified rutin, apigenin-7-*O*-glucoside, and luteolin-7-*O*-glucoside as the most abundant flavonoids in an aqueous extract of olive leaves from northeast Portugal. These results were recently corroborated by Makowska-Wąs and coworkers [31], who identified luteolin-7-*O*-glucoside, rutin, and apigenin-7-*O*-glucoside as the predominant flavonoids in wild olive leaves harvested in South Portugal. Regarding secoiridoids, oleuropein generally represents the most characteristic oleoside derivative in olive leaves. However, its content and bioconversion occur in different districts of the olive tree according to different factors, such as plant maturation, cultivar type, and harvest time [32]. Indeed, as observed in Table 1, although only OESA showed the presence of oleuropein (18.03%), OESY contained oleuropein hexoside (11.07%), as well as the highest content of hydroxyoleuropein isomer I and II (2.94% and 3.75% vs. 1.06% and 0.58% in OESA). On the contrary, OESY showed the highest content of tannins (19.96% vs. 1.97% in OESA), and it was a rich source of decaffeoylverbascoside (23.07%), absent in OESA. This phenyleptanoid is a derivative of verbascoside, a typical hydroxycinnamic derivative of olive fruit, generally only found in small amounts in olive leaves [31]. However, the amount of this metabolite in olive leaves may significantly vary depending on pedo-climatic conditions [32]. Furthermore, it seems that a reverse relationship exists between the oleuropein and verbascoside content [32], which could explain the absence of oleuropein in OESY. However, beyond the different varieties, it is well-known that biotic and abiotic stressors play a pivotal role in influencing the polyphenolic content of olive leaves, as well as the relative quantitative distribution of the main metabolites [33]. LC-DAD-ESI-MS analysis of OESA and OESY extracts identified different compounds that have an antimicrobial effect justified in the present study. The hydroalcoholic extracts from *O. europea* leaves were effective against *S. aureus* strains. As often reported with plant-derived extracts [34,35], the present study confirmed that Gram-positive strains were more susceptible compared with Gram-negative bacteria. Amongst the Gram-positive human pathogens, *S. aureus* and methicillin-resistant *S. aureus* (MRSA) play a crucial role, being responsible for several infections, including skin, respiratory, and bone joint infection, as well as endocarditis, bacteremia, and toxic shock syndrome [36]. Due to the increased spread of multi-drug resistance, more effort has been focused on novel antimicrobial agents against *S. aureus* and MRSA. According to many reports, phenolic compounds isolated from olive leaves have substantial antimicrobial activity [5,37,38]. The effectiveness of certain phenolic compounds in olive leaves, including caffeic acid, oleuropein, rutin, and verbascoside against *S. aureus*, has been reported [3,39]. Therefore, we hypothesize that these compounds, as well as potential synergistic effects within the extracts, were responsible for the activity reported here against *S. aureus*. The high relative proportion of decaffeoylverbascoside, a caffeoyl phenylethanoid glycoside in OESY, may be related to the stronger antimicrobial effect against *S. aureus* compared to OESA. Besides, olive leaf extract had significant antiviral activity against HSV-1 [17]. It has also been reported that olive leaf extracts exhibit antiviral activities against human immunodeficiency virus type 1 (HIV-1) [40,41]. *Olea europea* exhibits antiviral activity against viral hemorrhagic septicemia rhabdovirus (VHSV) [42], and is known to contain a mixture of polyphenolic compounds, including oleuropein, oleuropein aglycone, elenolic acid, and hydroxytyrosol [43]. Oleuropein possesses well-documented antiviral activity [17]; its efficacy against hemorrhagic septicemia rhabdovirus (VHSV), hepatitis B virus (HBV), and human immunodeficiency virus (HIV) was demonstrated [44]. The beneficial effect of oleuropein against VHSV is exerted through a virucidal effect, reducing virus infectivity and avoiding cell-to-cell fusion of uninfected cells [45]. Alternatively, the viral particle integrity could be damaged, as has been previously observed for the effect of flavonones on HSV-1 [46]. The data presented here indicate that both OESA and OESY exert a dose-dependent, broad-spectrum antiviral activity against HSV-1. However, the selectivity index (SI) was higher for OESY (SI between 4.1 and 7.4) compared to OESA (SI between 1.3 and 1.6). It is worth noting that, in addition to their anti-oxidative and anti-inflammatory characteristics, richness in polyphenols in OESA and OESY extracts can be the basis for anti-infective properties. Several extracts were proposed to interfere with HSV-1 attachment to cells via its surface structures and proteins, thereby blocking its adsorption and penetration into host cells.

HSV-1 entry is essential for the initiation, spread, and maintenance of viral infection, and represents the target for antiviral mechanisms exerted by OESA and OESY extracts. Indeed, our studies indicated that the time-of-addition assay and the inhibition entry assay were essential for the antiviral effect (Figure 4). Overall, our findings demonstrate that OESA and OESY interfere with the virus attachment to cell receptors, and thus reduce HSV-1 entry and replication on Vero cells.

## 5. Conclusions

In conclusion, the current study revealed that polyphenols from *O. europea* leaf extract exhibit an antiviral effect against HSV-1 and antimicrobial activity against *S. aureus*, which was stronger for the methicillin sensitive strains. Our findings demonstrated that *O. europea* leaf extracts, after one hour of viral adsorption, resulted in a significant neutralization of the virus in Vero cells. It is possible to hypothesize that one potential mechanism for this inhibitory effect of *O. europea* leaf extracts on the HSV-1 lytic cycle is the blocking of virion entry into the cells. However, there are still numerous questions that need to be answered, which will require further research. In particular, further studies are needed to explore the molecular mechanisms responsible for the antiviral activity, as well as the roles of polyphenols from *O. europea* leaves that may serve as a source of novel pharmacological treatment. Lastly, OESA and OESY extracts could be used in combination with other antimicrobial and antiviral drugs to reduce the emergence of resistant strains. 

This study suggests new strategies for manipulating HSV-1 infection both for prevention and therapeutic purposes.

## Figures and Tables

**Table 2:** MICs of OESY and OESA (expressed as µg/mL) against Gram-positive bacteria, Gram-negative bacteria, and the yeast *C. albicans*.

Strain	OESY	OESA
*E. coli* ATCC 10536	NA	NA
*S. aureus* ATCC 6538	7.81–15.62	15.62–31.25
*S. aureus* ATCC 43300	500–1000	1000
*P. aeruginosa* ATCC 9027	NA	NA
*C. albicans* ATCC 10231	NA	NA

MICs, minimal inhibitory concentrations; OESY, extract of *O. europea* var. sylvestris; OESA, extract of *O. europea* var. sativa. NA, not active.

**Table 3:** MICs of OESY and OESA (expressed as µg/mL) against clinical isolates of *S. aureus*.

Strain	OESY	OESA
*S. aureus* strain 3	NA	NA
*S. aureus* strain 6	1000	2000
*S. aureus* strain 8	NA	NA
*S. aureus* strain 14	NA	NA
*S. aureus* strain 26	NA	NA
*S. aureus* strain 32	NA	NA
*S. aureus* strain 84	NA	NA
*S. aureus* strain 526	NA	NA
*S. aureus* strain 531	NA	NA
*S. aureus* strain 550	NA	NA
*S. aureus* strain 808	1000	2000
*S. aureus* strain 814	NA	NA

MICs, minimal inhibitory concentrations; OESY, extract of *O. europea* var. sylvestris; OESA, extract of *O. europea* var. sativa. NA, not active.

**Table 4:** Selectivity index (SI), cytotoxicity (CC_50_), and antiviral activity (EC_50_) of OESA and OESY leaf extracts.

Extract	CC_50_ (mg/mL)	EC_50_ (mg/mL)	SI
**OESA**			
pre-infection	0.2	0.12	1.6
post-infection	0.2	0.15	1.3
**OESY**			
pre-infection	0.82	0.11	7.4
post-infection	0.82	0.2	4.1

CC_50_: half maximal cytotoxic concentration; EC_50_: half maximal effective concentration; SI: ratio of EC_50_/CC_50_; OESY, extract of *O. europea* var. *sylvestris*; OESA, extract of *O. europea* var. *sativa*.
